# Design, synthesis, and greener pasture biological assessment of a novel nucleoside: 1-(α-D-ribofuranosyl)-6,7-difluoro-2-methyl-4-quinazolinone as an inhibitor of COVID-19 and Alzheimer’s disease

**DOI:** 10.55730/1300-0527.3483

**Published:** 2022-06-16

**Authors:** Laila M. BREAK, Adil A. GOBOURI, Wafa S. Al-HSRTHI, Mohamed HAGAR, Nadia S. Al-KAFF, Musa A. SAID

**Affiliations:** 1 Department of Chemistry, College of Science, Taif University, Taif, Saudi Arabia; 2 Department of Chemistry, Faculty of Science, Alexandria University, Alexandria, Egypt; 3 Biology Department, College of Science, Taibah University, Al-Madinah Al-Munawara, Saudi Arabia; 4 Chemistry Department, College of Science, Taibah University, Al-Madinah Al-Munawara, Saudi Arabia; 5 Institute of Inorganic Chemistry, University of Stuttgart Pfaffenwaldring, Stuttgart, Germany

**Keywords:** Main protease, in silico, MD-DFT assessment, Alzheimer’s disease (AD), 6,7-difluoro-2-Methyl-4-quinazolinone

## Abstract

Synthesis of a new fluorinated nucleoside of 6,7-difluoro-2-methyl-4-quinazolinone was described. 2-Amino-4,5-difluorobenzoic acid **1** reacts with (CH_3_CO)_2_O followed by ammonia to form *(1H)-*6,7-difluoro-2-methyl-4-quinazolinone **3a**. Ribosylation of a silylated **4** with l-*O*-acety1-2,3-5-tri-*O*-benzoyl-α-D-ribofuranose **5** forms a protected nucleoside **6** then unprotected from **6** to give a free nucleoside **7**. Greener pasture biological docking of the cystine protease of COVID-19 [M^pro^, code 7BQY, PDB] by novel nucleoside and fluoroquinazoline compounds is presented. LIGPLOT (2D) representations calculated for the same ligands are shown. A superposition of remdesivir approved medicine, **N3** inhibitor, and our ligands docked together into the binding protein of 7BQY is also given for a fair comparison. The binding affinities of remdesivir, **N3** inhibitor, the nucleoside **7**, and fluoroquinazoline **3a**, **3b** compounds with **7**BQY calculated under the same conditions are −7.7, −7.4, −7.6, −6.1, and −6.1 kcal mol^−1^, respectively. The high values were due to the existence of many hydrophobic interactions and hydrogen bonds between the ligands and the active amino acid residues of the receptor, indicating a promising candidate as a COVID-19 inhibitor. Pro Tox -II server showed that compound **7** has a similar feature to the approved antiviral drug remdesivir for COVID-19. Additionally, a fascinating molecular modeling investigation showed that our nucleoside demonstrated good binding inhibition of AChE enzyme towards advancing an efficient medication against Alzheimer’s disease. Finally, DFT has been conducted to illustrate the MD results in terms of the molecular descriptor-based structural activity relationship calculated from FMOs.

## 1. Introduction

4-quinazolinones are solids and stable to alkaline treatment and mild acid. The most common approach to synthesizing 4(*3H*)-quinazolinone products is the direct reaction of 2-aminobenzoic acid (anthranilic acid) derivatives with acetic anhydride to give the benzoxazinone, followed by condensation with nitrogen nucleophiles [1–4]. The 2-methyl group in *3H* or *1H*-4-quinazolinone is more responsive than 4-quinazoline. In general, quinazolinones have several biological applications such as preventing cancer [2,5,6], anti-HIV [7], cytotoxicity in vitro [8], antiinflammatory [6,9], antibacterial [10,11], antifungal [12], and antiviral properties [13,14]. Furthermore, the kinase inhibitory potency of many N-aryl thiazolo[5,4-f]quinazolin-4-amines has been revealed, intending to improve the healing of Down syndrome (DS) and early AD [15–18]. The fluorine atom is a significant substituent in medicinal chemistry due to its electronic properties and improved molecular lipophilicity [19]. New fluorinated hydroquinazoline derivatives were used as antifungal agents [20] and anticancer agents [21]. A difluorinated inhibitor showed 4.23 times larger potency against the epidermal growth factor receptor (EGFR) than a nonfluorinated inhibitor [22]mutations of which have been linked specifically to nonsmall-cell lung cancer. For the L858R/T790M/C797S triplet mutant (EGFRTM. In addition, quinazolinone nucleosides [23–26] and fluorinated nucleosides have been used extensively in biological activity such as the prevention of cancer cells from forming, anti-HCV activity in vitro [27], and anti-HBV agents [28].

This article aims to report a new fluorinated nucleoside quinazolinone containing two fluorine atoms at positions 6 and 7 of the quinazolinone moiety [29]. When this compound was reacted with l-*O*-acety1-2,3-5-tri-*O*-benzoyl-*β*-D-ribofuranose **5** by silated method [30], the fluorinated nucleoside; 6,7-difluoro 2-methylquinazolin-4-one **7** was obtained. Also, given the current several ongoing concerns in medicine finding to monitor the horrific effect of the virus on our daily lives [3,31–33], we have been encouraged to screen, in silico as a greener pasture preliminary step, the interaction between our novel nucleoside: 1-(2,3,5-tri-O-benzoyl-*β*-D-ribofuranosyl)-6,7-difluoro-2-methyl-4-quinazolinone, **7** and fluoroquinazoline, **3** ligands with the main protease (downloaded from RCSB, PDB) deposition code 7BQY) active site as possible candidate drugs for COVID-19. It is worth mentioning here that nucleoside was tested before, in vitro, for antiviral activity against two representative cowpox viruses, ortho poxviruses, and vaccinia virus [34]. We also investigated, in silco, the molecular modeling of our nucleoside as a binding inhibition of AChE enzyme towards the advancement of an efficient drug against Alzheimer’s disease.

## 2. Materials and methods

TLC was done using silica gel 60 (aluminum sheet, Fluka company) and revealed by UV-vis light. Melting points (mp) of all synthesized compounds were determined using an electrothermal device and are uncorrected. The ^1^H and ^13^C NMR spectra were measured on an NMR spectrometer in CDCl_3_, CD_3_OD at 213 and 850 MHz. Mass spectra were measured on GC MS-QP 2000 EX mass spectrometer at 70 e.V (King Abdel Aziz University).

### 2.1. Synthesis

**Synthesis of 6,7-difluoro-2-methylbenzo[2,3-d]oxazin-4-one (2)**2-amino-4,5-difluorobenzoic acid **1** (1.6416 g, 0,009 mol) with a suitable quantity of acetic anhydride was refluxed for 1 h to reveal compound **2** as a brown powder. Yield: 1.6971 g (89.5%); mp: 158 °C; molecular formula: C_9_H_5_NO_2_F_2_; molecular weight (mol. wt): 197.13.

**Synthesis of 1H-6,7-difluoro-2-methyl-4-quinazolinone (3a)**1.69 g (0.009 mol) of compound **2** with a suitable quantitative amount of ammonia solution was refluxed for 6 h, cooled, and then treated with acetic acid to give crystals collected by filtration as a white powder of compound **3a**. Yield: 0.3690 g (21.95%); mp: 263–270 °C; molecular formula: C_9_H_6_F_2_N_2_O, mol. wt: 196.15, MASS m/z (%)**:** M^+^= 196.06 (100%), 196.04 (100%) [29,35].

#### Ribosylation of 1H-6,7-difluoro-2-methyl-4-quinazolinone

**1-(2,3,5-tri-O-benzoyl-**β**-D-ribofuranosyl)-6,7-difluoro-2-methyl-4-quinazolinone (6)**A mixture of 1H-6,7-difluoro-2-methyl-4-quinazolinone **3a** (0.369 g, 0.0018 mol), dry HMDS (20 mL) and a catalytic quantity of (NH_4_)_2_SO_4_ were heated under reflux for 24 h (TLC). The product was evaporated to dryness in anhydrous media to afford the silylated derivative **4** as an intermediate compound, which added (10 mL) of dry 1,2-dichloroethane, (0.4694 g, 0.9 mmol) 1-*O*-acetyl-2,3,5-tri-O-benzoyl-*β*-D-ribofuranose (**5)** and (2 mL, 10 mmol) trimethylsilyl triflate; (TMSOTf as a catalyst). The obtained solution was stirred for 14 days at room temperature and then washed with an aqueous NaHCO_3_ followed by water and dried over sodium sulfate. The silica gel column chromatography with ethyl acetate and chloroform (2:98) was used in order to separate the pure product. After evaporation, the main fraction **6** was obtained as a sticky yellow material. Yield: 0.378 g (49.93%); mp: 180 °C; molecular formula: C_35_H_26_F_2_N_2_O_8_; mol. wt: 640.59; ^1^HNMR (CDCl_3_, 850 MHz, TMS), δ H: 8.06–7.13 (m, 17H Aromatic protons), 6.0–6.1 (d, 1H, *J* = 4.25 Hz, H-1′), 5.62 (t, *J* = Hz,1H, H-2′), 4.97 (t, *J* = Hz, 1H, H-3′), 4.49–4.72 (m, 1H, H-5′), 4.01–4.06 (m, 1H, H-4′), 1.56 (s, 3H) CH_3_); ^13^CNMR (CD_3_OD, 213 MHz, TMS), δ: 190.00, 167.63, 167.32, 164.32, 157.06, 155.73, 141.90, 138.05, 132.93, 132.87, 132.83, 130.57, 130.54, 129.71, 130.59, 129.92, 129.77, 129.74, 129.72, 129.50, 128.66, 128.43, 128.42, 128.40, 128.32, 128.29, 119.10; 117.75, 101.43, 79.16, 75.76, 70.25, 60.32, 65.14 CH_2_, 17.65 CH_3_.

#### Deprotection of 1-(2,3,5-tri-O-benzoyl-α-D-ribofuranosyl)-6,7-difluoro-2-methyl-4-quinazolinone-1-(α-D-ribofuranosyl)-6,7-difluoro-2-methyl-4-quinazolinone (7)

A mixture of compound **6** (0.5 mmol), and sodium metal (0.001g) in methanol (dry, 10 mL), 0.04 mmol) was stirred at room temperature for 1day. It was then treated with drops of acetic acid to neutralize the solution. The residue was recrystallized from water to offer **7** as light-yellow crystals. Yield: 0.152 g (81.28%); mp: 255 °C; molecular formula: C_14_H_14_F_2_N_2_O_5_, mol. wt: 328.27; ^1^HNMR (CD_3_OD, 850 MHz, TMS), δ: 7.88 (s, 1H, H-5′), 6.94 (s, 1H, H-8), 6.9 (d, 1H, *J* = 6.80 Hz, H-1′), 6.60–6.63 (m, 1H, H-2′), 5.65 (d, 1H, *J* = 4.25 Hz, OH2′), 5.33 (d, 1H, *J* = 1.7 Hz, OH3′), 5.18 (d, 1H, *J* = 3.4 Hz, OH5′), 4.70–4.95 (m, 3H, H-3′, OH-3′, OH-2′), 3.63–3.68 (m, 2H, H-4′, OH-4′), 3.52(t, *J*_1_ = 6.80, *J*_2_ = 6.80 Hz, 1H, H-5′), 1.88 (s, 3H, CH_3_); ^13^CNMR (CD_3_OD, 213 MHz, TMS), δ: 160.0, 129.3, 128.8, 128.2, 127.2, 127.1, 126.0, 124.0, 78.0, 61.6, 51.2, 51.0, 50.9, 29.3; MASS m/z (%): M^+^ = 327.21, 326.00, 322.24, 319.18 (100), 306.24, 301.10, 298.21, 293.98, 287.08, 279.07, 263.12, 260.05, 328.14, 252.20, 243.94, 232.96, 220.02, 203.96, 193.11, 190.90, 184.95, 171.95, 163.98, 155.16, 142.95, 123.99, 107.03, 102.90, 91.97. (calc. 328.27).

### 2.2. Docking in silico studies

The molecular docking studies of our nucleoside compound and donepezil were done using the PyRx 0.8 (https://sourceforge.net/projects/pyrx/). It is a recommended and powerful visualization engine [36]offering preprocessing and postprocessing adapted (to date. The settings in the PyRx 0.8 include: Grid box (19.61, 29.01, 36.27 A˚), (3.54, 64.29, 64.01 A˚) centered at (19.61, 29.10, 26.27), (23.06, 25.0, 25.0) for 7BQY and 1ELE. Energy range = 4 and exhaustiveness = 8. Water molecules and the N3 ligand were deleted from the proteases (PDB code 7BQY, 1EVE). The key residues of 7BQY used in this study were identified before [31, 37].

### 2.3. Molecular descriptor-based structural activity relationship calculated from FMOs

The optimal architectural structures of the synthesized compounds **3a**, **3b**, and **7** were computed in the gas phase using Gaussian 9 on the B3LYP 6-311G basis set and have been used in the calculation of the molecular chemical descriptors. The energy levels of the frontier molecular orbitals highest occupied molecular orbitals (HOMOs) and lowest occupied molecular orbitals (LUMOs) could be used to compute several chemical descriptors (least unoccupied molecular orbitals). Furthermore, HOMOs and LUMOs in the examined compounds could be used as a qualitative predictor of their ability to donate or receive electrons from the neighboring receptor [38–40]. FMOs, in general, are a powerful component for obtaining realistic qualitative data on excitation qualities in a variety of chemical and pharmacological processes [39,41–44]. Furthermore, FMO-derived chemical descriptors have been employed to estimate biological activities [45–54]4-dihydro-[1,2,4]triazole-3-thione was synthesized and structurally characterized by elemental analysis, FT-IR, Raman, ^1^H and ^13^C-NMR and UV–Vis studies. A density functional theory (DFT. Likewise, the FMOs energy levels and the energy gaps may influence the kind and amount of binding during their interactions with receptors. As a result, nonbonding intermolecular interactions such as hydrophilic interactions and H-bonding occur with the receptor.

Table 1 presents many estimated thermodynamic molecular descriptors, including dipole moment (μ), electronegativity (χ), charge transfer prevention extent, global hardness (η), and electrophilicity (*ω*) determined from electronegativity and chemical hardness values.

## 3. Results and discussion

**Scheme 1:**
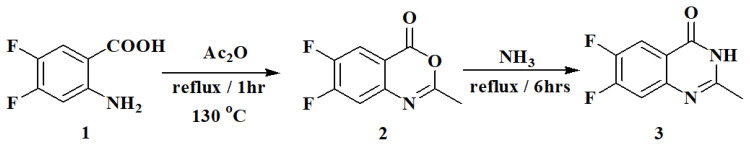
Synthesis of nucleoside (*3H*)-6,7-difluoro-2-methyl-4-quinazolinone

Compounds **2**–**7** were prepared as displayed in Schemes 1 and 3. The structures of the newly synthesized organic compounds were confirmed using ^1^H, ^13^C NMR, and mass spectra.

Benzoxazinone compounds can be prepared by treatment of anthranilic acid derivatives with acid chloride [1] or acetic anhydride [2,55]5-dimethyl-2-thiazolyl. The 6,7-difluoro-4-benzoxazinone **2** was prepared from the reaction of 2-amino-4,5-difluorobenoic acid **1** with acetic anhydride for 1 h. Treatment of compound **2** with ammonia solution for 6 h afforded the (*3H*)-6,7-difluoro-2-methyl-4-quinazolinone **3**, Scheme 1.

**Scheme 2:**
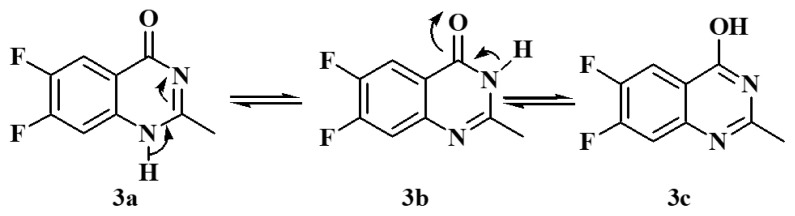
Tautomeric properties of (1H)-6,7-difluoro-2-methyl-4-quinazolinone **3a**,**3b**, and **3c**.

Weddige identified the tautomeric characteristics of (3*H*) 4-quinazolinones, which could exist in three tautomeric forms. The existence of 4-hydroxy quinazoline was displayed by its stability in aqueous alkali at pH 12 to give the anion form. The 4-quinazolinones usually do not dissolve in alkali, mainly when a substitute is present on N1 or N3 [15]. The compound, 6,7-difluoro-2-methyl-4-quinazolinone is expected to have three tautomeric forms of (*1H*) 6,7-difluoro-2-methyl-4-quinazolinone **3a, 3b**, and **3c**, Scheme 2.

**Figure 1:**
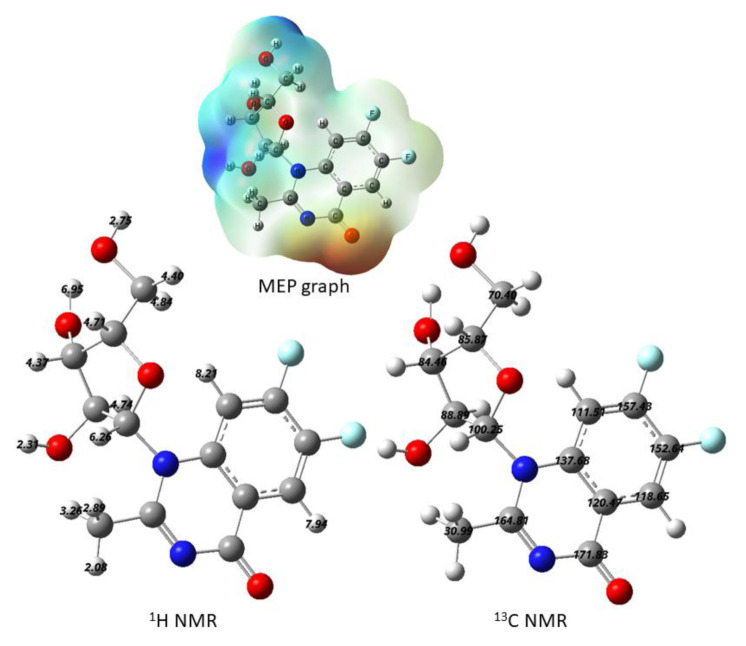
Calculated ^1^H and ^13^C NMR of an optimized molecular geometry of compound **7** in deuterated methanol solvent. MEP graph is also presented.

**Scheme 3:**
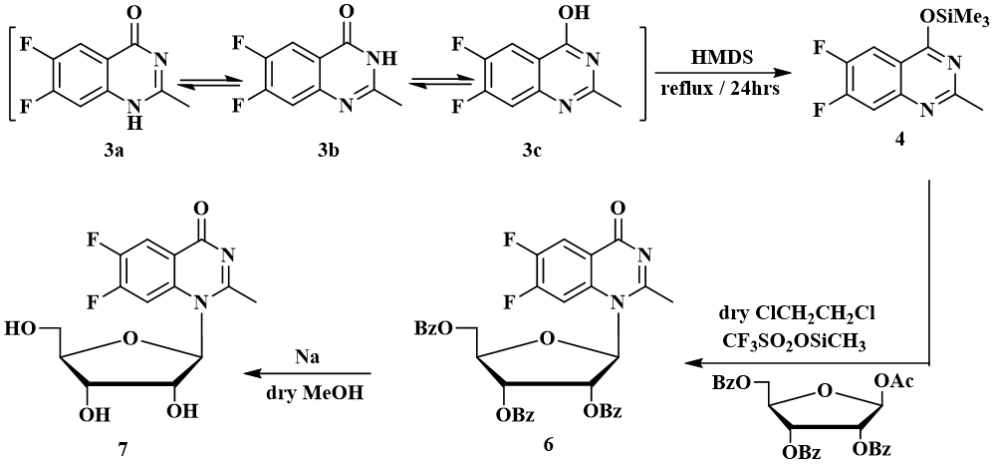
Synthesis of nucleoside 6,7-difluoro-2-methyl-4-quinazolinone.

A mixture of **3a**, dry HMDS, and a catalytic quantity of (NH_4_)_2_SO_4_ was heated under reflux for 24 h (TLC). The product was evaporated to dryness in anhydrous media to afford the silylated derivative **4** as an intermediate compound. Compound **5** was treated with trimethylsilyl triflate; (TMSOTf as a catalyst), an aqueous NaHCO_3,_ and silica gel column chromatography with ethyl acetate and chloroform (2:98) to obtain **6** as a sticky yellow material. The ^1^H NMR of the protected nucleoside **6** shows a doublet signal at δ = 6.1 ppm allocated to the anomeric protons of the ribose moiety with a *J* coupling constant equal to 4.25 Hz matches the 1′-proton. The spectra appeared as multiple signals of the configuration at δ = 8.06–7.13 ppm due to benzoyl groups and quinazolinone protons in an aromatic region, see Scheme 3 and Figure 1.

Deprotection of the benzoyl group of protected nucleoside **6** was achieved in sodium metal in dry methanol at room temperature for 24 h to give the corresponding free nucleoside **7**. The ^1^H NMR of **7** showed the prospective base moiety protons and the sugar moiety protons, though no signal for benzoyl protons appeared. Also, the ^1^H NMR spectrum of **7** shows a doublet at δ = 6.9 ppm allocated to the anomeric proton of the ribose moiety with *J* coupling constant equal to 4.25 Hz that matches the 1′-proton the *β*-configuration. With the appearance of aromatic group complex signals, a singlet peak appeared at δ = 7.88 ppm allocated to the H-5, and another singlet signal at δ = 6.94 ppm assigned to the H-8. The ^1^H NMR of **7** showed the predicted base moiety protons in addition to the sugar moiety protons. Calculated ^1^H, and ^13^C NMR of an optimized molecular geometry of compound **7** in deuterated methanol solvent are provided for comparison (Figure 1; experimental section). Compound **7** was confirmed using mass spectra which showed a molecular ion peak ion (M^+^) at m/z = 327.21 (2.77%), (calc. 328.27) for molecular formula C_14_H_14_F_2_N_2_O_5_, the base peak = 319.18 (100).

### 3.1. DFT theoretical calculations

#### 3.1.1. Molecular geometry

**Figure 2:**
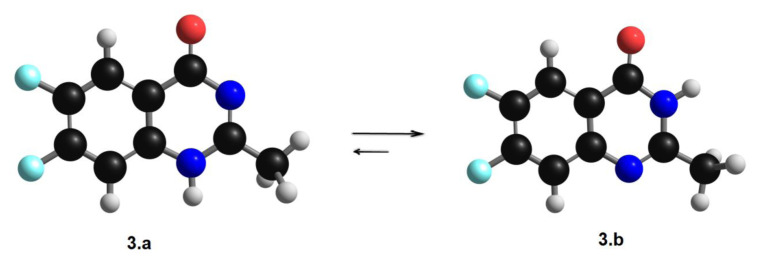
Calculated optimized molecular geometry of both isomers of compound **3**.

The optimal molecular structures were predicted using DFT calculations at the B3LYP 6311G (d, p) basis set to assess the stability of the expected positional isomers. To estimate the most stable positional isomer of the produced compound **3**, calculations were performed for the proposed two isomers, **3a** and **3b**. These computations entailed doing a geometry structure optimization on each isomer to obtain the least energy structure, then calculating the frequency at the optimized geometry. In addition, several thermochemical parameters were calculated (Figure 2; Table 2). Free energy (G) and enthalpy (H) were calculated to determine the relative stabilities, corrected energy, and thermodynamic parameters of both positional isomers of compound **3**.

**Figure 3:**
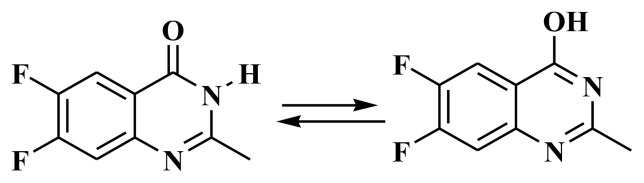
Tautomeric isomerism of compound **3**.

The DFT calculations revealed that the **3b** isomer had the lowest energy structure and the most stability in comparing both geometrical isomers. The **3b** isomer, on the other hand, is the least stable. The energy difference between both isomers is 11.2 kcal/mol. However, the amid derivative **3b** higher stability could be illustrated in its tautomeric ability with the conjugated C=O group (Figure 3).

**Figure 4:**
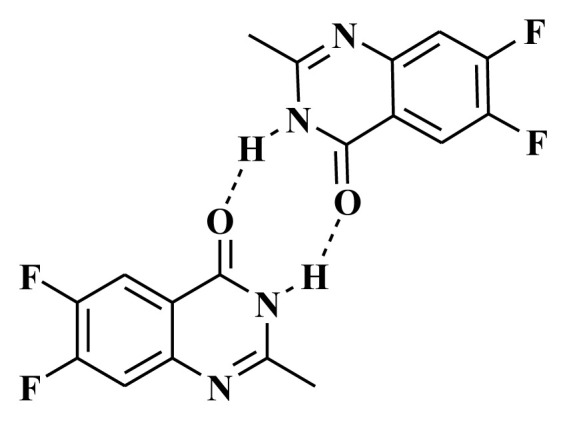
Intermolecular H-bonding of amid isomer **3b**.

On the other hand, the intermolecular H-bonding could affect the stability of the predicted stable isomer **3b**. Isomer **3b** can form intermolecular H-bonding with two strong H-bonds than the other isomer **3a**. The formation of two H-bonds will enhance the stability of the amid isomer **3b** more than the other isomer (Figure 4).

### 3.2. Docking analysis

**Figure 5:**
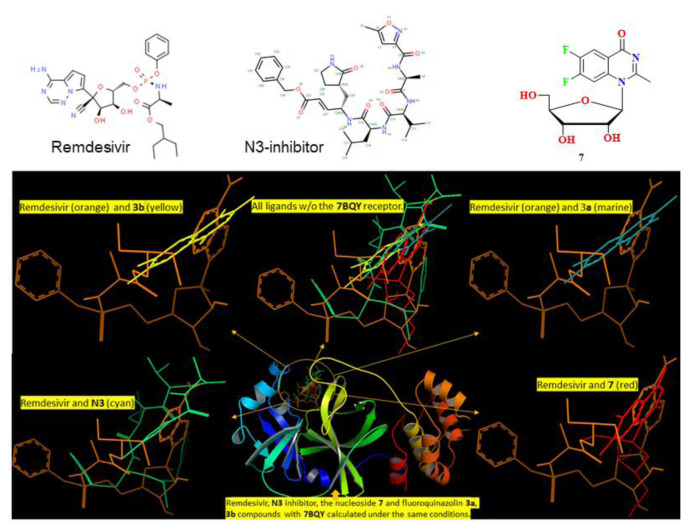
The superposition of compounds **3a**, **3b**, **7**, remdesivir drug, and **N3** inhibitor docked into the binding pocket of 7BQY using the identical parameters for a fair comparison (circled, middle bottom window). For clarity, all docked ligands are shown in the top central window without the 7BQY, M^pro^. Finally, the display of each ligand after docking is compared to remdesivir (orange in color). This is represented by PyMOL by Schrödinger (downloaded from https://pymol.org/2/) [56].

**Figure 6:**
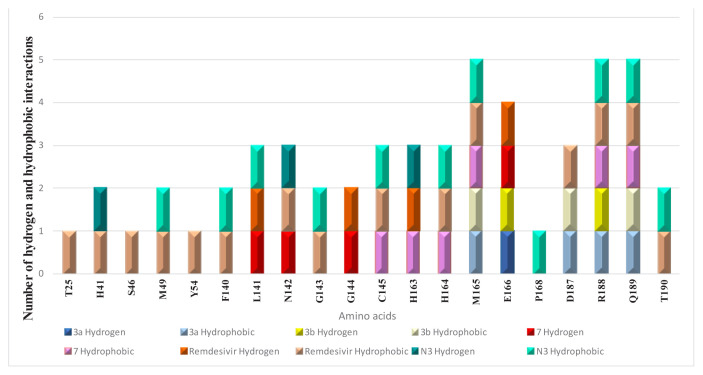
Number and type of interactions between the remdesivir, N3, 7, 3a, 3b, and the main protease (7BQY).

**Figure 7:**
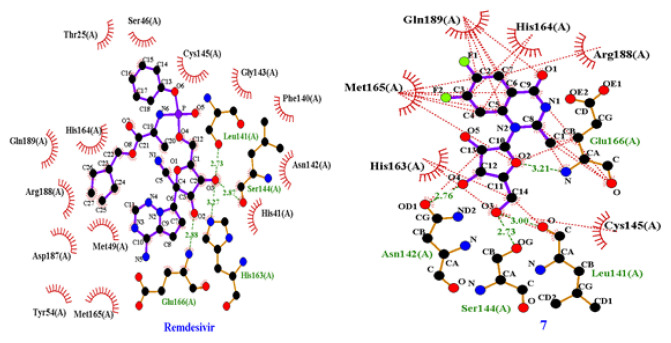
(a) A schematic 2D LIGPLOT illustration of remdesivir and the main protease 7BQY. The purple stick at the center represents remdesivir, whereas the remaining are the amino acids residues. The green dashes are the hydrogen bond, and the thin red dashes and the spoked arcs pointing towards the ligand represent the hydrophobic residue bonds with remdesivir. The active site residues: Leu141(A), Asn143(A), Ser144(A), and Glu166(A) are involved in making a hydrogen bond with the ligand. Atoms marked by spokes in the ligand or protein are involved in the interactions without the red lines. (b) A schematic 2D LIGPLOT illustration of compounds **7** and 7BQY represented by PyMOL [56].

This docking study investigates how a nucleoside ligand might interact in the active site of the main protease (M^pro^; PDB code 7BQY, 1EVE) for Alzheimer’s disease and COVID-19 [15]. Hydrogen bonding, hydrophobic interactions, and other factors, e.g., entropy and solvation, can control the structural reorganization of both the ligand and the receptor upon binding. It remains challenging to predict the conformational changes of 7BQY, 1EVE, and the ligand, as both are expected to display different degrees of adjustment after binding [33]. The docked molecule in 7BQY is shown in Figure 5. The confirmation of all molecules has been demonstrated with respect to the known medicine Remdesivir Figure 5. It has been noticed that the Met165(A), Arg188(A), Gln189(A), and Gly143(A) amino acids are the common residues, forming hydrophobic interactions among all the compounds used in this study. At the same time, Remdesivir forms four hydrogen bonds (Leu141(A), Asn143(A), Ser144(A) and Glu166(A)). Similarly, compound **7** also forms 4 hydrogen bonds ((Leu141(A), Ser144(A), His163(A) and Glu166(A)). **N3** displays three hydrogen bonds (Leu41(A), Asn142(A) and His163(A)). Compounds **3a** and **3b** show one and two hydrogen bonds, respectively, Figures 6 and 7. A superposition of compounds **3a**, **3b**, **7**, remdesivir drug, and **N3** inhibitor docked into the binding pocket of 7BQY using the identical parameters for a fair comparison. The outcomes are presented in Figure 5. The number and type of interactions between the remdesivir, **N3**, **7**, **3a**, **3b** and the main protease (7BQY) are summarized in Figure 6 and partly in Figure 7. The laydown of all docked ligands is presented together (with and without 7BQY), showing their fitting in the same active site but different positioning (Figure 5). Also, the display of the superposition of each ligand compared to remdesivir is displayed for easy comparison. This is represented by PyMOL by Schrödinger [56].

### 3.3. Binding prediction of compound 7 compared to donepezil using molecular docking

**Figure 8:**
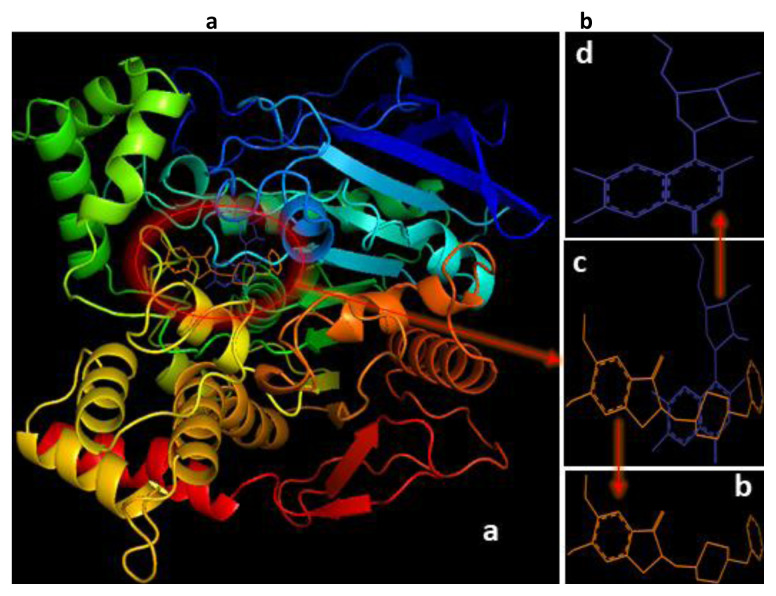
(a) The superposition of compound **7** and donepezil docked to **1EVE** using the identical parameter for a reasonable comparison; (b) the display of donepezil drug after docking; (c) the display of both donepezil drug and compound **7**; (d) the display of compound **7**. Results are presented using PyMOL [56].

**Figure 9:**
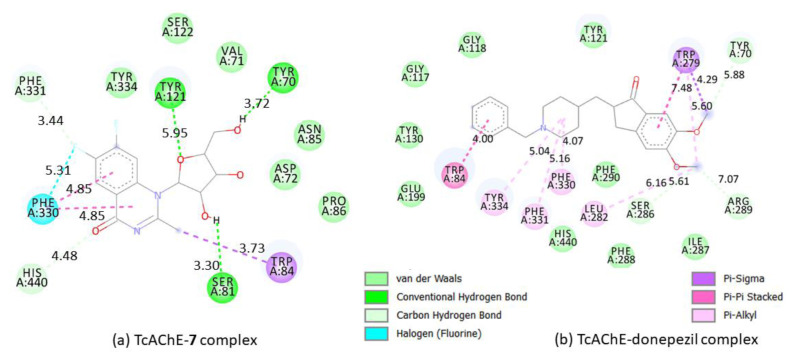
2D representations of interactions in (a) TcAChE- **7** complex; (b) in TcAChE-donepezil complex. Distances are in A^o^.

Docking analysis of compound **7** and donepezil was carried out on the Torpedo California acetylcholinesterase to study their binding affinity (TcAChE) (PDB, 1EVE), [57–62]. Compound **7** demonstrated a close-fitting binding against the TcAChE enzyme with close binding energy of −9.7 kcal/mol compared to donepezil (−10.9 kcal/mol) (Figures 8 and 9). The molecular structure of **7** and donepezil after binding with the protein are shown in Figures 8 and 9. Compound **7** shows conventional hydrogen bonds, fluorine-hydrogen bonds, carbon-hydrogen bonds, and van der Waals interaction. In contrast, the donepezil drug does not exhibit any of these interactions, indicating probably considerable flexibility of compound **7** that facilitates the tight interaction with the binding site for the quaternary nitrogen of AChE enzyme [33]. Donepezil drug demonstrates mainly p-s and p-p stacked interactions (Figure 9). Superimposition of **7** (blue line) and donepezil (orange line) is shown in Figure 8, which demonstrates the benzylpiperidine group oriented towards PHE, TYR, and TRP residues. Similarly, the quinazoline moiety in compound **7** is directed towards PHE, TYR, and TRP residues. The inden-1-one group of donepezil oriented towards TRP, ARG, and LEU residues (Figure 9), whereas the hydrofuran moiety in **7** is directed towards SER, TYR, and Val residues. TRP84 interacts via π-sigma interaction with a distance of 3.37 Å. In contrast, in the case of donepezil, it exhibits a π - π stacking with a distance of 4 Å (Figure 9). Three conventional hydrogen bonds were found between the hydroxyl groups and SER81, TYR70, and TYR121 residues with distances of 3.3, 3.72, and 5.95 Å, respectively (Figure 9). Interestingly, as can be seen, at the bottom of the gorge, a C=O···HIS440 hydrophobic interaction was formed in the active site of AChE [63]. This interaction was not detected in the donepezil drug case. This might enhance the affinity to the enzyme and therefore improve the inhibition effect of compound **7**.

### 3.4. Toxicity prediction (in silico) for our ligands in comparison to remdesivir and N3

ProTox-II virtual lab was used to predict the toxicity of our small molecules in comparison to the authorized drug remdesivir [64–70] and **N3** inhibitor [31]. The oral toxicity presented as lethal dose (LD) at 50% (LD50) milligrams per kilograms weight of the test population. ProTox-II predicted the toxicity classes as class 3 for **3a** and **3b** and LD50 of 200 mg/kg with the same average similarity and prediction accuracy of 60.65% and 68.07%, respectively (Table 3). Interestingly, the toxic activity of 7 is predicted as class 4, which is similar to the approved drug, remdesivir, with LD50 of 1000 mg/kg. The average similarity and prediction accuracy of 7 and remdesivir were 66.03%, 68.07%, and 40.93%, 54.26%, respectively (Table 3). Compound N3 was predicted as the lowest toxic compound in this study as class 5 with LD50 of 4000 mg/kg and average similarity and prediction accuracy of 45.06% and 54.26, respectively (Table 3).

The ProTox-II web server can also predict organ toxicity. For example, the hepatoxicity estimation of the three ligands **3a**, **3b**, the approved medicine for COVID-19 remdesivir and **N3** inhibitor were all not active. In contrast, **7** was predicted as a functional ligand on organ toxicity (Lever) with a probability of 0.52. Predicted activities for all studied ligands and the controls (remdesivir and **N3**) were inactive (noncarcinogenic, non immunotoxin, nonmutagenic, noncytotoxic) (Table 4).

**Figure 10:**
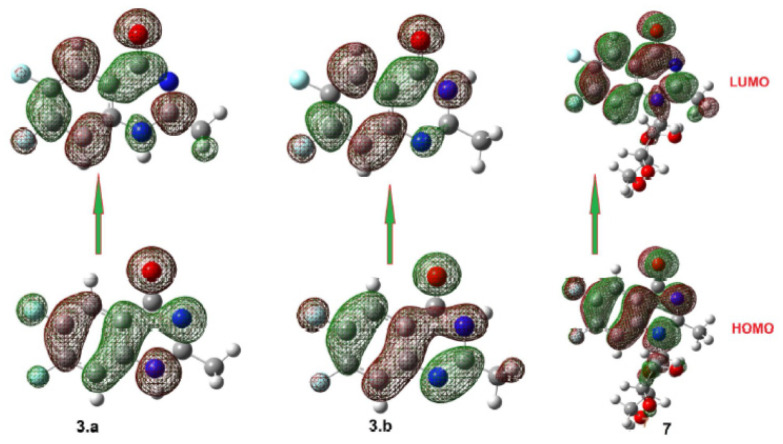
Molecular orbital distribution and localization for FMOs of **3a, 3b**, and **7**.

Figure 10 depicts the prediction of the FMOs energy levels and their energy gap of **3a** and **3b** and its nucleoside **7**. The energy levels of the FMOs in compound **3a** are lower than those in compound **3b**, which could be explained in terms of conjugation. The presence of an H-atom adjacent to the C=O group enhances the tautomerism with the OH. It may decrease the conjugation of the C=O group with the ring, inhibiting the conjugation with the benzene ring. This effect on MO levels has an impact on the chemical descriptors that are used to illustrate the biological activity of these compounds. However, the attachment of the hydrophilic carbohydrate moiety of the isomer **3a** affects the energy difference between the orbitals with a small value ΔE = 4.74 e.V. Also, it is evident that the isomerism highly affects the level of the LUMO than the HOMO. Isomer **3b** shows the lowest-lying LUMO. The low-lying LUMO orbital of **3b** could predict its ability to accept the electron than its tautomer and its nucleoside derivative **7**. However, the higher topological, polar surface area, lower hydrophobicity, in silico absorption and high H-bonding acceptor percent of compound **7** could illustrate its high predicted biological activity against the inhibition of COVID 19 and Alzheimer infections.

## 4. Conclusions

Synthesis of some of the ribosylation of silated compound **4** with 1-*O*-acetyl-2,3,5-tri-*O*-benzoyl-*β*-D-ribofuranose **5** gave *β*-anomeric of the benzoylated nucleoside derivatives **6**. Deprotection of the latter using dry absolute methanol and sodium metal gave the new free N-nucleosides **7**, in moderate yields. The new compounds obtained have been characterized by their spectral analysis. The prediction analysis using the Pro Tox -II server showed that compound **7** has a similar feature to the approved antiviral drug remdesivir for COVID-19. Compound **7** behaved similarly in all tests except on hepatotoxicity. This suggests that compound **7** may be worth additional study in the context of a possible drug for COVID-19. A molecular modeling investigation confirmed, in silco, that our nucleosides **7** is an excellent binding inhibition of AChE enzyme. Compound **7** could be a possible effective drug against Alzheimer’s disease. Finally, DFT was used to demonstrate molecular geometry and the thermodynamic parameters that could be used to illustrate the MD results in terms of the structural activity correlation computed from FMOs using molecular descriptors. Compound **7** demonstrated its predicted activity towards binding inhibition of AChE enzyme and Alzheimer’s disease due to higher hydrophilicity, a larger topological polar surface area, and a strong H-bonding acceptor.

## Figures and Tables

**Table 1:** Dipole moment and chemical reactivity descriptors (Debye, μ) of the prepared compounds 3 and 7.

Parameter	3a	3b	7
LUMO	−2.17	−1.97	−2.23
HOMO	−6.96	−6.96	−6.96
ΔE_LUMO-HOMO_	4.80	5.00	4.74
$\chi =-\frac{1}{2}(E_{HOMO}+E_{LUMO})$	4.57	4.47	4.60
$\eta =-\frac{1}{2}(E_{HOMO}-E_{LUMO})$	2.40	2.50	2.37
$\delta =\frac{1}{\eta }$	0.42	0.40	0.42
$\omega =\frac{\chi^{2}}{2\eta }$	4.34	3.99	4.46
I=-E_HOMO_	6.96	6.96	6.96
A=-E_LUMO_	2.17	1.97	2.23
μ	4.87	5.87	6.87

**Table 2:** Thermal parameters (hartree/particle) of both positional isomers of compound **3**.

Parameter	3a	3b
E_corr_	0.139237	0.139796
ZPVE	−730.840481	−730.858147
E*_tot_*	−730.829206	−730.847069
H	−730.828262	−730.846124
G	−730.878053	−730.895234
ΔE in kcal/mol	11.209	0.00

**Table 3:** Acute oral toxicity predicted by ProTox-II web server for ligands **3a, 3b, 7**, remdesivir, and **N3**.

Ligands drug and N3	Oral toxicity prediction results			
	Predicted LD50 (mg/kg)	Predicted toxicity class	Average similarity (%)	Prediction accuracy (%)
3a	200	3	60.65	68.07
3b	200	3	60.65	68.07
7	1000	4	66.03	68.07
Remdesivir	1000	4	40.93	54.26
N3	4000	5	45.06	54.26

**Table 4:** Organ toxicity and toxicological endpoints predicted activity calculated using the ProTox-II web server for **ligands 3a, 3b, 7**, remdesivir, and **N3**.

Ligands drug and N3	Hepatotoxicity		Carcinogenicity		Immunotoxicity		Mutagenicity		Cytotoxicity	
	Activity	Probability	Activity	Probability	Activity	Probability	Activity	Probability	Activity	Probability
**3a**	x	0.54	x	0.51	x	0.99	x	0.64	x	0.90
**3b**	x	0.54	x	0.51	x	0.97	x	0.64	x	0.90
**7**	√	0.52	x	0.66	x	0.96	x	0.77	x	0.77
Remdesivir	x	0.56	x	0.55	x	0.90	x	0.62	x	0.55
**N3**	x	0.58	x	0.52	x	0.96	x	0.61	x	0.67

## References

[1] Santagati NA Bousquet E Spadaro A Ronsisvalle G 4-Quinazolinones: Synthesis and reduction of prostaglandin E2 production Farmaco 1999 54 780 784 10.1016/S0014-827X(99)00102-0 10668179

[2] Zayed MF Ahmed HEA Ihmaid S Omar ASM Abdelrahim AS Synthesis and screening of somenew fluorinated quinazolinone-sulphonamide hybrids as anticancer agents Journal of Taibah University Medical Sciences 2015 10 333 339 10.1016/j.jtumed.2015.02.007

[3] Singhal TA Review of Coronavirus Disease-2019 (COVID-19) Indian Journal of Pediatrics 2020 87 281 286 10.1007/S12098-020-03263-6 32166607 PMC7090728

[4] Radwan AA Alanazi FK Biological Activity of Quinazolinones IntechOpen 2020 10.5772/intechopen.90621

[5] Poorirani S Sadeghian-Rizi S Khodarahmi G Khajouei M Hassanzadeh F Synthesis and cytotoxic evaluation of novel quinazolinone derivatives as potential anticancer agents Research in Pharmaceutical Sciences 2018 13 450 10.4103/1735-5362.236838 30271447 PMC6082030

[6] Gawad N Georgey H Youssef R El-Sayed N Synthesis and antitumor activity of some 2, 3-disubstituted quinazolin-4 (3H)-ones and 4,6-disubstituted-1, 2,3,4-tetrahydroquinazolin-2H-ones European Journal of Medicinal Chemistry 2010 45 6058 6067 21051122 10.1016/j.ejmech.2010.10.008

[7] Andreeva OV Belenok MG Saifina LF Shulaeva MM Dobrynin AB Synthesis of novel 1,2,3-triazolyl nucleoside analogues bearing uracil, 6-methyluracil, 3,6-dimethyluracil, thymine, and quinazoline-2,4-dione moieties Tetrahedron Letters 2019 60

[8] Abdel Gawad NM Georgey HH Youssef RM El-Sayed NA Synthesis and antitumor activity of some 2, 3-disubstituted quinazolin-4(3H)-ones and 4,6-disubstituted-1,2,3,4-tetrahydroquinazolin-2H-ones European Journal of Medicinal Chemistry 2010 45 6058 6067 10.1016/j.ejmech.2010.10.008 21051122

[9] Rajput C Singhal S Synthesis, characterization, and antiinflammatory activity of newer quinazolinone analogs, Downloads.Hindawi.Com 2013 Journal of pharmaceutics 2013 2013 1 7 10.1155/2013/907525 PMC4590821 26556002

[10] Gatadi S Lakshmi TV Nanduri S 4(3H)-Quinazolinone derivatives: Promising antibacterial drug leads European Journal of Medicinal Chemistry 2019 170 157 172 10.1016/J.EJMECH.2019.03.018 30884322

[11] Sharaf A Ragab S Elbarbary A Elkhabiry S Synthesis and biological evaluation of some 3H-quinazolin-4-one derivatives Springer 2021 10.1007/s13738-021-02315-8

[12] Zhang J Liu J Ma Y Ren D Cheng P One-pot synthesis and antifungal activity against plant pathogens of quinazolinone derivatives containing an amide moiety Bioorganic Medicinal Chemistry Letters 2016 26 2273 2277 10.1016/J.BMCL.2016.03.052 27040656

[13] Asif M Chemical Characteristics, Synthetic Methods, and Biological Potential of Quinazoline and Quinazolinone Derivatives International Journal of Medicinal Chemistry 2014 2014 1 27 10.1155/2014/395637 PMC4321853 25692041

[14] Demeunynck M Baussanne I Survey of Recent Literature Related to the Biologically Active 4(3H)-Quinazolinones Containing Fused Heterocycles Current Medicinal Chemistry 2013 20 794 814 10.2174/0929867311320060006 23276134

[15] Zahlenreilien L Lage D Ueber I Beitrage zur Kenntniss Journal Für Praktische Chemie 1887 141 154 (in German).

[16] Foucourt A Hédou D Dubouilh-Benard C Girard A Taverne T Design and Synthesis of Thiazolo[5,4-f]quinazolines as DYRK1A Inhibitors, Part I Molecules 2014 19 15546 15571 25268714 10.3390/molecules191015546 PMC6270991

[17] Foucourt A Hédou D Dubouilh-Benard C Girard A Taverne T Design and synthesis of thiazolo[5,4-f]quinazolines as DYRK1A inhibitors, Part II Molecules 2014 19 15411 15439 10.3390/molecules191015411 25264830 PMC6271009

[18] Couly F Diharce J Bonnet P Meijer L Fruit C Besson T Conception of DYRK1A kinase inhibitors via metal-catalyzed C–H arylation, inspired by fragment-growing studies 4th International Electronic Conference on Medicinal Chemistry 2018 5580 10.3390/ecmc-4-05580

[19] Shah P Westwell AD The role of fluorine in medicinal chemistry Journal of Enzyme Inhibition and Medicinal Chemistry 2007 22 527 540 10.1080/14756360701425014 18035820

[20] Ghorab MM Abdel-Gawad SM El-Gaby MSA Synthesis and evaluation of some new fluorinated hydroquinazoline derivatives as antifungal agents Farmaco 2000 55 249 255 10.1016/S0014-827X(00)00029-X 10966155

[21] Layeva AA Nosova EV Lipunova GN Trashakhova TV Charushin VN A new approach to fluorinated 4(3H)-quinazolinones Journal of Fluorine Chemistry 2007 128 748 754 10.1016/j.jfluchem.2007.03.005

[22] Akher FB Farrokhzadeh A Ravenscroft N Kuttel MM Mechanistic Study of Potent Fluorinated EGFR Kinase Inhibitors with a Quinazoline Scaffold against L858R/T790M/C797S Resistance Mutation: Unveiling the Fluorine Substituent Cooperativity Effect on the Inhibitory Activity Journal of Physical Chemistry B 2020 124 5813 5824 10.1021/ACS.JPCB.0C03440 32603111

[23] Break LM Alharthi W Synthesis new of nucleoside of 1, 3-bis-(2,3,5-tri-O-benzoyl-β-D-ribofuranosyl)-8-(trifluoromethyl)-2-methyl-4-quinazolinone Proceedings 2018 9 57 10.3390/ecsoc-22-05694

[24] Break LM Synthesis of 8-Trifluloromethyl-2-Thioquinazolin-(3H)-4-One Nucleosides International Journal Chemistry 2017 9 82 10.5539/ijc.v9n4p82

[25] Break LM Synthesis and Characterization of New 8-trifluloromethyl Quinazolin-2,4-(3H)-Dione Nucleosides International Journal Chemistry 2017 9 73 10.5539/ijc.v9n1p73

[26] Break LM Synthesis of Some of Fluorinated Benzimidazole Nucleosides International Journal Chemistry 2016 8 188 10.5539/ijc.v8n1p188

[27] Ouahrouch A Taourirte M Engels JW Benjelloun S Lazrek HB Synthesis of new 1,2,3-triazol-4-yl-quinazoline nucleoside and acyclonucleoside analogues Molecules 2014 19 3638 3653 10.3390/molecules19033638 24662079 PMC6271638

[28] Qiu J Chen W Zhang Y Zhou Q Chen J Assessment ofquinazolinone derivatives as novel non-nucleoside hepatitis B virus inhibitors European Journal of Medicinal Chemistry 2019 176 41 49 10.1016/J.EJMECH.2019.05.014 31091479

[29] Laeva AA Nosova EV Lipunova GN Golovchenko AV Adonin NY Fluorine-containing heterocycles: XIX. synthesis of fluorine-containing quinazolin-4-ones from 3,1-benzoxazin-4-ones Russian Journal of Organic Chemistry 2009 45 913 920 10.1134/S1070428009060190

[30] Vorbrüggen H Krolikiewicz K Bennua B Nucleoside syntheses, XXII Nucleoside synthesis with trimethylsilyl triflate and perchlorate as catalysts Chemische Berichte 1981 114 1234 1255 10.1002/cber.19811140404

[31] Jin Z Du X Xu Y Deng Y Liu M Structure of Mpro from SARS-CoV-2 and discovery of its inhibitors Nature 2020 582 289 293 10.1038/s41586-020-2223-y 32272481

[32] Dhama K Sharun K Tiwari R Dadar M Singh Malik Y COVID-19, an emerging coronavirus infection: advances and prospects in designing and developing vaccines, immunotherapeutics, and therapeutics HumanVaccines & Immunotherapeutics 2020 16 1232 1238 10.1080/21645515.2020.1735227 PMC7103671 32186952

[33] Alsafi MA Hughes DL Said MA First COVID-19 molecular docking with a chalcone-based compound: Synthesis, single-crystal structure and Hirshfeld surface analysis study Acta Crystallographics Secttion: C Structural Chemistry 2020 76 1043 1050 10.1107/S2053229620014217 33273140

[34] Fan X Zhang X Zhou L Keith KA Kern ER A pyrimidine-pyrazolone nucleoside chimera with potent in vitro anti-orthopoxvirus activity Bioorganic & Medicinal Chemistry Letters 2006 16 3224 3228 10.1016/j.bmcl.2006.03.043 16603351

[35] Wang CX Zhang ZL Yin QK Tu JL Wang JE Design, Synthesis, and Evaluation of New Quinazolinone Derivatives that Inhibit Bloom Syndrome Protein (BLM) Helicase, Trigger DNA Damage at the Telomere Region, and Synergize with PARP Inhibitors Journal Medicinal Chemistry 2020 63 9752 9772 10.1021/ACS.JMEDCHEM.0C00917/SUPPL_FILE/JM0C00917_SI_002.CSV 32697083

[36] Allouche A Khodair AI El-Barbary AA Imam DR Kheder NA Software News and Updates Gabedit — A Graphical user interface for computational Chemistry softwares Journal of Computational Chemistry 2012 32 174 182 10.1002/jcc 20607691

[37] Wallace AC Laskowski RA Thornton JM Ligplot: A program to generate schematic diagrams of protein-ligand interactions Protein Engineering Design and Selection 1995 8 127 134 10.1093/protein/8.2.127 7630882

[38] Al-Otaibi JS Sheena Mary Y Shyma Mary Y Panicker CY Thomas R Cocrystals of pyrazinamide with p-toluenesulfonic and ferulic acids: DFT investigations and molecular docking studies Journal Molecular Structure 2019 1175 916 926 10.1016/J.MOLSTRUC.2018.08.055

[39] Hagar M Ahmed HA Aljohani G Alhaddad OA Investigation of some antiviral N-heterocycles as COVID 19 drug: Molecular docking and DFT calculations International Journal of Molecular Sciences 2020 21 3922 10.3390/ijms21113922 32486229 PMC7312990

[40] Pap C Mohapatra RK El-ajaily MM Alassbaly FS Sarangi AK DFT, anticancer, antioxidant and molecular docking investigations of some ternary Ni (II) complexes with 2-phenol Springer 2021 75 1005 1019 10.1007/s11696-020-01342-8

[41] Bouachrine M Hamidi M Bouzzine SM Taoufik H Theoritical study on the structure and electronic properties of new materials based on thiophene and oxadiazole Journal of Applied Chemical Research 2009 10 29 37 https://www.sid.ir/en/Journal/ViewPaper.aspx?ID=195555

[42] Yang L Feng JK Liao YAM Ren QM Theoretical studies on the electronic and optical properties of two blue-emitting fluorene-pyridine-based copolymers Optical Materials 2007 29 642 650 10.1016/j.optmat.2005.11.024

[43] Alnoman RB Parveen S Hagar M Ahmed HA Knight JG A new chiral boron-dipyrromethene (BODIPY)-based fluorescent probe: molecular docking, DFT, antibacterial and antioxidant approaches Journal Biomolecular Structure and Dynamis 2020 38 5429 5442 10.1080/07391102.2019.1701555. 31809642

[44] Alnoman RB Hagar M Parveen S Ahmed HA Knight JG Computational and molecular docking approaches of a New axially chiral BODIPY fluorescent dye Journal of Photochemistry and Photobiology A: Chemistry 2020 395 112508 10.1016/j.jphotochem.2020.112508

[45] Joshi R Pandey N Yadav SK Tilak R Mishra H Synthesis, spectroscopic characterization, DFT studies and antifungal activity of (E)-4-amino-5-[N′-(2-nitro-benzylidene)-hydrazino]-2,4-dihydro-[1,2,4]triazole-3-thione Journal Molecular Structure 2018 1164 386 403 10.1016/j.molstruc.2018.03.081

[46] Joshi R Kumari A Singh K Mishra H Pokharia S Triorganotin(IV) complexes of Schiff base derived from 1,2,4-triazole moiety: Synthesis, spectroscopic investigation, DFT studies, antifungal activity and molecular docking studies Journal Molecular Structure 2020 1206 127639 10.1016/j.molstruc.2019.127639

[47] Khodair AI Awad MK Gesson JP Elshaier YAM New N-ribosides and N-mannosides of Rhodamine Derivatives for Suppressing Leukemia Cell Line Growth Journal Clinical Haematology 2020 1 7 9 10.33696/haematology.1.002

[48] Suresh Kumar S Athimoolam S Sridhar B Structural, spectral, theoretical and anticancer studies on new co-crystal of the drug 5-fluorouracil Journal Molecular Structure 2018 1173 951 958 10.1016/j.molstruc.2018.07.079

[49] Hagar M Ahmed HA El-Sayed TH Alnoman R Mesophase behavior and DFT conformational analysis of new symmetrical diester chalcone liquid crystals Journal of Molecular Liquids 2019 285 96 105 10.1016/j.molliq.2019.04.083

[50] Grover M Singh B Bakshi M Singh S Quantitative structure-property relationships in pharmaceutical research - Part 2 Pharmaceutical Science and Technology Today 2000 3 50 57 10.1016/S1461-5347(99)00215-1 10664573

[51] Malhotra R Ravesh A Singh V Synthesis, characterization, antimicrobial activities, and QSAR studies of organotin(IV) complexes Phosphorus, Sulfur and Silicon Related Elements 2017 192 73 80 10.1080/10426507.2016.1225054

[52] Kumer A Sarker MdN Paul S The simulating study of HOMO, LUMO, thermophysical and quantitative structure of activity relationship (QSAR) of some anticancer active ionic liquids Eurasian Journal of Environmental Research 2019 3 1 10 https://dergipark.org.tr/en/pub/ejere/478362

[53] Lewis DFV Quantitative structure-activity relationships (QSARs) within the cytochrome P450 system: QSARs describing substrate binding, inhibition and induction of P450s Inflammopharmacology 2003 11 43 73 10.1163/156856003321547112 15035734

[54] Almehmadi MA Aljuhani A Alraqa SY Ali I Rezki N Aouad MR Hagar M Design, synthesis, DNA binding, modeling, anticancer studies and DFT calculations of Schiff bases tethering benzothiazole-1,2,3-triazole conjugates Journal Molecular Structure 2021 1225 129148 10.1016/j.molstruc.2020.129148

[55] Jiang JB Hesson DP Dusak BA Dexter DL Kang GJ Synthesis and biological evaluation of 2-styrylquinazolin-4(3H)-ones, a new class of antimitotic anticancer agents which inhibit tubulin polymerization Jounral Medicinal Chemistry 1990 33 1721 1728 https://pubs.acs.org/doi/pdf/10.1021/jm00168a029 10.1021/jm00168a029 2088342

[56] DeLano WL PyMOL Reference Guide Delano Sci San Carlos, CA, US 2004 1 68

[57] Sulimov VB Kutov DC Taschilova AS Ilin IS Tyrtyshnikov EE Sulimov AV Docking Paradigm in Drug Design Current Topics in Medicinal Chemistry 2021 21 6 507 546 10.2174/1568026620666201207095626 33292135

[58] Pant S Singh M Ravichandiran V Murty USN Hemant Kumar Srivastava HK Peptide-like and small-molecule inhibitors against Covid-19 Journal of Biomolecular Structure and Dynamics 2021 May 39 8 2904 2913 10.1080/07391102.2020.1757510 32306822 PMC7212534

[59] Sonmez F Kurt BZ Gazioglu I Basile L Dag A Cappello V Ginex T Kucukislamoglu M Sonmez F Kurt BZ Gazioglu I Basile L Cappello V Ginex T Kucukislamoglu M Guccione S Design, synthesis and docking study of novel coumarin ligands as potential selective acetylcholinesterase inhibitors Journal Enzyme Inhibition and Medicinal Chemistry 2017 32 1 285 297 10.1080/14756366.2016.1250753 PMC6010140 28097911

[60] Karakaya S Koca M Volkan Yılmaz S Yıldırım K Pınar NM Molecular docking studies of coumarins isolated from extracts and essential oils of zosima absinthifolia link as potential inhibitors for alzheimer’s disease Molecules 2019 24 722 10.3390/molecules24040722. 30781573 PMC6412260

[61] Bajda M Wiȩckowska A Hebda M Guzior N Sotriffer CA Structure-based search for new inhibitors of cholinesterases International Journal of Molecular Sciences 2013 14 5608 5632 10.3390/ijms14035608 23478436 PMC3634507

[62] Qin J Lan W Liu Z Huang J Tang H Synthesis and biological evaluation of 1, 3-dihydroxyxanthone mannich base derivatives as anticholinesterase agents Chemistry Central Journal 2013 7 https://www.sciencedirect.com/science/article/pii/S0022286020314708 accessed November 25, 2021 10.1186/1752-153X-7-78 PMC3673835 23622085

[63] Brown AD Clegg MT Kahler AL Weir BS Chang TT Policy Statement on Cooperative Research and Development Agreements and Intellectual Property Licensing, Sci Technol 1990 167 22

[64] Peele KA Potla Durthi C Srihansa T Krupanidhi S Ayyagari VS Molecular docking and dynamic simulations for antiviral compounds against SARS-CoV-2: A computational study Informatics in Medicine Unlocked 2020 19 100345 10.1016/j.imu.2020.100345 32395606 PMC7211761

[65] Gavriatopoulou M Ntanasis-Stathopoulos I Korompoki E Fotiou D Migkou M Emerging treatment strategies for COVID-19 infection Clinical and Experimental Medicine 2021 21 2 167 179 10.1007/s10238-020-00671-y 33128197 PMC7598940

[66] Rosenberg K Remdesivir in The Treatment of COVID-19 American Journal of Nursing 2021 Jan 1 121 1 55 10.1097/01.NAJ.0000731668.01845.8c 33350698

[67] Yousefi H Mashouri L Okpechi SC Alahari N Alahari SK Repurposing existing drugs for the treatment of COVID-19/SARS-CoV-2 infection: A review describing drug mechanisms of action Biochemical Pharmacology 2021 183 114296 10.1016/j.bcp.2020.114296 33191206 PMC7581400

[68] Mhatre S Naik Sh Patravale V A molecular docking study of EGCG and theaflavin digallate with the druggable targets of SARS-CoV-2 Computers in Biology and Medicine 2021 Feb 129 104137 10.1016/j.compbiomed.2020.104137 33302163 PMC7682485

[69] Crampon K Giorkallos A Deldossi M Stéphanie Baud, Luiz Angelo Steffenel, Machine-learning methods for ligand-protein molecular docking Drug Discovery Today 2022 Jan 27 1 151 164 10.1016/j.drudis.2021.09.007 34560276

[70] Talluri S Molecular Docking and Virtual Screening Based Prediction of Drugs for COVID-19 Combinatorial Chemistry & High Throughput Screening 2021 24 5 716 728 10.2174/1386207323666200814132149 32798373

